# Ewing's Sarcoma of the Head and Neck: A Retrospective Analysis of 24 Cases

**DOI:** 10.1080/13577149977811

**Published:** 1999-03

**Authors:** Ayman Allam, Gamal El-Husseiny, Yasser Khafaga, Alaa Kandil, Alan Gray, Adnan Ezzat, Henrik Schultz

**Affiliations:** 1 Department of Radiation Oncology King Faisal Specialist Hospital and Research Center PO Box 3354 Riyadh 11211 Saudi Arabia; 2 Department of Medical Oncology King Faisal Specialist Hospital and Research Center Riyadh Saudi Arabia

## Abstract

*Introduction and purpose*. Primary Ewing's sarcoma arising from the bones of the head and neck region is extremely rare representing only 1– 4% of all Ewing's sarcoma cases. Previous reports suggest a better prognosis for that particular anatomic site. The purpose of this study was to analyze the clinico-epidemiologic characteristics of that rare clinical presentation, as well as its patterns of failure and prognosis following treatment.

*Materials and methods.* This study included a retrospective review of the medical records of patients with the diagnosis of Ewing's sarcoma of the head and neck region treated at King Faisal Specialist Hospital and Research Center between 1975 and 1996.

*Results*. Out of a total number of 24 cases analyzed, there were 17 males and 7 females with a ratio of 2.4:1. The median age at diagnosis was 16.5 years. A painful swelling was the most common clinical presentation.The maxilla was the most common site of presentation (9/24 cases). There were 3/24 cases who presented with metastatic disease at diagnosis.The majority of patients (16/24 cases) had a tumor size >10 cm. Most patients were treated with systemic chemotherapy plus localized irradiation following an initial biopsy.With a mean follow up of 3.4 years, the 5-year actual overall survival (OS) for the whole group was 53%, while the 5-year actuarial disease-free survival (DFS) was 30%. These figures were higher than those repor ted from our institution for young patients (≤ 14 years treated for Ewing' s sarcoma in other anatomic locations (30% v 15%). The response to chemotherapy was the only prognostic factor that affected both the OS and DFS.

*Conclusion.*  The prognosis of Ewing's sarcoma of the head and neck region is slightly better than that of other anatomic sites.The response to systemic chemotherapy is one of the most important prognostic factors affecting both DFS and OS of Ewing's sarcoma of the head and neck. Multimodality therapy consisting of an initial biopsy, aggressive combination chemotherapy and localized radiotherapy is the treatment of choice for Ewing's sarcoma of the head and neck region and may result in long-term survival.


*Sarcoma* (1999) 3, 11–15

ORIGINAL ARTICLE

### Ewing's sarcoma of the head and neck: a retrospective analysis of 24 cases*

AYMAN ALLAM,[1] GAMAL EL-HUSSEINY,[1] YASSER KHAFAGA,[1] ALAA KANDIL,[1] ALAN GRAY,[1] ADNAN EZZAT[2] & HENRIK SCHULTZ[1]

[1]*Departments of Radiation Oncology, and* [2]*Medical Oncology, King Faisal Specialist Hospital and Research Center, Riyadh, Kingdom of Saudi Arabia*

**Abstract**

*Introduction and purpose.* Primary Ewing's sarcoma arising from the bones of the head and neck region is extremely rare representing only 1–4% of all Ewing's sarcoma cases. Previous reports suggest a better prognosis for that particular anatomic site. The purpose of this study was to analyze the clinico-epidemiologic characteristics of that rare clinical presentation, as well as its patterns of failure and prognosis following treatment.

*Materials and methods.* This study included a retrospective review of the medical records of patients with the diagnosis of Ewing's sarcoma of the head and neck region treated at King Faisal Specialist Hospital and Research Center between 1975 and 1996.

*Results.* Out of a total number of 24 cases analyzed, there were 17 males and 7 females with a ratio of 2.4:1. The median age at diagnosis was 16.5 years. A painful swelling was the most common clinical presentation. The maxilla was the most common site of presentation (9/24 cases). There were 3/24 cases who presented with metastatic disease at diagnosis. The majority of patients (16/24 cases) had a tumor size >10 cm. Most patients were treated with systemic chemotherapy plus localized irradiation following an initial biopsy. With a mean follow up of 3.4 years, the 5-year actuarial overall survival (OS) for the whole group was 53%, while the 5-year actuarial disease-free survival (DFS) was 30%. These figures were higher than those reported from our institution for young patients (≤14 years treated for Ewing's sarcoma in other anatomic locations (30% v 15%). The response to chemotherapy was the only prognostic factor that affected both the OS and DFS.

*Conclusion.* The prognosis of Ewing's sarcoma of the head and neck region is slightly better than that of other anatomic sites. The response to systemic chemotherapy is one of the most important prognostic factors affecting both DFS and OS of Ewing's sarcoma of the head and neck. Multimodality therapy consisting of an initial biopsy, aggressive combination chemotherapy and localized radiotherapy is the treatment of choice for Ewing's sarcoma of the head and neck region and may result in long-term survival.

### Introduction

Primary Ewing's sarcoma arising in the head and neck region is extremely rare, comprising 1–4% of all cases of Ewing's sarcoma.[1,2] Most authors claim a better prognosis for Ewing's sarcoma of the head and neck region as compared to that arising in other anatomic locations.[1–5] The most commonly affected bones in the head and neck region are the skull, the mandible and the maxilla.[1] There has also been case reports of localized Ewing's sarcoma affecting the orbital roof,[3] the retropharynx[4] and the nasal cavity.[5] The aim of the present study was to review the cases of Ewing's sarcoma of the head and neck region treated at our institution during the past 20 years, aiming at a better understanding of the clinico-epidemiologic characteristics of that rare anatomic location. The details of treatment including surgery, chemotherapy and radiation therapy, as well as the patterns of failure are presented. Also, the various potential prognostic factors affecting both the disease-free (DFS) and the overall survival (OS) are highlighted.

### Materials and methods

The following study included a retrospective analysis of data available from the medical records of 24 cases of primary Ewing's sarcoma arising in the head and neck region, treated at the King Faisal Specialist Hospital and Research Center during the period 1975 to 1996. The King Faisal Specialist Hospital and Research Center is a tertiary care center serving the

Correspondence to: Dr Henrik Schultz, Department of Radiation Oncology, MBC 34, King Faisal Specialist Hospital & RC, PO Box 3354, Riyadh 11211, Kingdom of Saudi Arabia. Fax: 9661-442 4566.

*This paper has been accepted as an oral presentation in the 17th Annual ESTRO meeting, Edinburgh, 20–24 September 1998.

1357-714X/99/010011-05 $9.00 © 1999 Taylor & Francis Ltd whole kingdom of Saudi Arabia. It is the largest oncology referral center at present receiving patients from all over the kingdom.

The analysis included reviewing various prognostic factors that could affect the treatment outcome in terms of DFS as well as OS. The charts were reviewed for age, sex, presenting symptoms and signs and duration of symptoms. The primary tumor site was determined for each patient. The tumor size as well as the initial stage of presentation were determined for all patients. Staging work-up of patients including computerized tomography (CT) scan of the head and neck, chest X-ray, CT scan of the chest, bone scan, bone marrow biopsy and serum lactic dehydrogenase (LDH) levels were reviewed.

The different treatment modalities adopted including surgery, radiotherapy and chemotherapy were reviewed in detail. The radiation therapy charts were reviewed for the total dose given, the number of fractions, the overall treatment time, the energy used and the use of CT planing. Chemotherapy details included timing of systemic chemotherapy, agents used, number of cycles as well as the response to chemotherapy treatment.

Patients were followed up regularly every 3 months for the first year, every 6 months in the subsequent 2 years and then yearly thereafter. Patients who were lost to follow-up were censored from the survival analysis at the time of last follow-up. None of those patients participated in clinical trials.

The patterns of treatment failure, whether local recurrence (LR) or distant metastasis (DM), were studied in detail. Dates of local recurrence and distant metastasis, as well as the initial site of DM were determined. Both actuarial OS and DFS were analyzed using the Kaplan–Meier method.

### Results

Out of 24 evaluable patients, there were 17 males and 7 females with a male to female ratio of 2.4:1. The median age of the patients was 16.5 years (range 2–33 years). A painful swelling was the most common presentation found in more than 90% of patients as shown in Table 1. The mean duration of symptoms was 5.5 months (range 2–12 months).

The maxilla was the most common site of presentation (9/24 cases). Metastasis at initial presentation was found in only 3/24 cases. Most of our patients had tumor size >10 cm in diameter (16/24 cases).

A bone marrow core biopsy from the iliac crest was performed in 19 patients; and was found to be infiltrated by malignant cells in 1 patient only. The LDH was assayed in 19 patients and was elevated in 12/19 (63%). A bone scan was done in 22/24 patients (92%). It showed localized increased uptake opposite the initial site of bony involvement in all 22 patients with no evidence of metastatic spread.

The majority of patients had biopsy only (16/24 cases); while the remaining had either incomplete excision (6/24 cases) or complete surgical excision (2/24 cases).

**Table 1**. *Characteristics of evaluable patients*

| Patients' characteristics | Number of cases (%) |
|---|---|
| 1. Sex | |
| Male | 17/24 (71%) |
| Female | 7/24 (29%) |
| 2. Symptoms | |
| Swelling | 23/24 (96%) |
| Pain and Tenderness | 21/24 (87.5%) |
| Visual problems | 8/24 (33%) |
| Anemia | 8/24 (33%) |
| Fever | 3/24 (12.5%) |
| 3. Tumor size | |
| 5–10 cm | 8/24 (33%) |
| >10 cm | 16/24 (67%) |
| 4. Initial stage | |
| Localized | 21/24 (87.5%) |
| Metastatic | 3/24 (12.5%) |
| 5. Tumor site | |
| Maxilla | 9/24 (37.5%) |
| Mandible | 6/24 (25%) |
| Orbit | 4/24 (17%) |
| Skull | 3/24 (12.5%) |
| Nasal Cavity | 2/24 (8%) |

The most common treatment modality was initial biopsy followed by combined systemic chemotherapy plus radiotherapy to the primary site involved. This modality was adopted in 14/24 cases (58%). There were various other treatment modalities, the most common of which was surgical excision followed by post-operative chemo-radiotherapy in 5/24 cases (21%).

Systemic chemotherapy was delivered in 22/24 cases (92%). Two patients refused systemic chemotherapy treatment, 1 was treated with complete surgical excision followed by post-operative radiotherapy while the other was treated with incomplete excision and refused post-operative irradiation. The most common regimen given was VAC (vincristine, adriamycin, cyclophosphamide; 10 patients) or VAC alternating with IEP (ifosfamide, etoposide and cisplatinum) given in 7 patients and VAIA (vincristine, adriamycin, ifosfamide and actinomycin D) in 5 patients. The choice of chemotherapy regimen depended on the protocol adopted during a given period of time, i.e. patients treated from 1975 to 1980 received VAC alone, while those treated from 1980 to 1990 received VAC/IEP. All patients treated from 1990 onwards received VAIA. There were only 13/22 patients (59%) who completed six or more cycles of systemic chemotherapy. Complete remission was obtained in 57% of patients, while partial remission occurred in 43% of patients. None of the patients had disease progression on chemotherapy.

Radiotherapy was delivered in 20/24 cases (83%), with a radical intent in 19/20 patients (95%). The total radiation dose ranged from 3000 to 5600 cGy with a median dose of 5040 cGy. There were 3/19 patients who received a total radiation dose ≤4000 cGy.

The total dose of radiation given depended on the treating physician. It did change to a more radical dose (5400 cGy) over time. The majority of the patients (13/20) were treated with 6 MV energy photons. Six patients were treated with Cobalt 60 and the remaining patient with 8 MV energy photons. CT planing was used in 10/20 cases (50%).

There were 7/24 cases who developed LR (29%), and 11/24 cases (46%) who developed DM. The lungs were the most common single site of DM (27%); there were 4/11 patients (36%) who had more than one site of DM. Five out of 24 patients developed both LR and DM.

With a mean follow-up time of 3.4 years, the 5-year actuarial OS for the whole group of patients was 53%; while the actuarial 5-year DFS was 30% (Fig. 1a and b). There was no statistically significant difference in 5-year OS and DFS between males and females (54% vs 50% and 26% vs 36%, respectively). Tumor size did not seem to have any impact on OS and DFS. The actuarial 5-year DFS for patients with tumor size of 5–10 cm was 30% compared to 29% for those with tumors >10 cm in diameter ($p = 0.93$).

Analysis of the survival results (both DFS and OS) according to the primary tumor site did not show any significant impact of that factor on the treatment outcome. The mandible had the highest 5-year OS (80%), followed by the maxilla, skull and orbit (71%, 50% and 38%, respectively). This difference was not statistically significant.

The response to chemotherapy treatment was the only prognostic factor which affected both the OS and DFS. The 5-year OS for patients with complete remission (CR) was 68% compared to 0% for patients with partial remission (PR) to chemotherapy ($p = 0.015$); also the 5-year DFS for CR patients was significantly higher than that of PR patients (27% vs 0%; $p = 0.02$).

### Discussion

Ewing's sarcomas arising from the bones of the head and neck region are extremely rare. Most cases in the literature are reported sporadically as case reports. The largest reported series until now is that of the Intergroup Ewing's Sarcoma Study (IESS) in 1987. Siegal *et al.*[1] reported in that series on a total of 29 cases of Ewing's sarcoma of the head and neck region which represented 4% of all Ewing's sarcoma cases reported by the IESS group. In the present series, Ewing's sarcoma of head and neck constituted 9% of all Ewing's cases treated in this institute (24/259 cases). This relatively high figure might be attributed to the fact that our hospital is the main referral oncology center in the region. The male to female ratio was 2.4:1, this is in accordance with the literature being a disease where males are more frequently affected.[6] The median age at diagnosis for this study was 16.5 years, which represents an expected figure for Ewing's sarcoma, a disease affecting primarily patients <20 years of age.[7]

The maxilla was the most common site of presentation in this study (9/24 cases). Although this represents a rare site of primary presentation, yet there has been a total of 22 cases of primary Ewing's sarcoma of the maxilla reported in the literature.[8,9]

In the present series, most patients were treated with initial biopsy followed by systemic chemotherapy plus radical radiotherapy. This is due to the fact that most of our patients have large lesions (>10 cm) of the head and neck, where radical surgery would be mutilating. Radical surgery can, however, play an important role in achieving local control in certain areas (e.g. mandible and skull), especially in tumors <5 cm in diameter where the treatment related morbidity would not be increased.[5,10,11]

The local control rate in the present study was 71%. This figure is lower than the 90% figure reported in other series.[12] There are two main factors affecting the local control rate, these are the initial tumor size and the total radiation dose delivered. The median dose in this study (5040 cGy) is considered adequate to achieve local control by most investigators.[12,13] Of the 3 patients who received a total radiation dose ≤4000 cGy, only 1 developed local recurrence. The lower local control rate figure obtained in the present study seems to be attributed to the large tumor size (>10 cm) in the majority of our patients (16/24 cases). Most of our patients present relatively late in the course of their disease due to long distances, cultural reasons and lack of efficient primary care hospitals in remote areas of the kingdom.

The 5-year actuarial DFS as well as OS in the present study (30% and 53%, respectively) are lower than the figures reported by IESS (80% survival at 3 years). Again we attribute those lower figures mainly to two factors: the larger tumor size at initial presentation as well as the low compliance rate to chemotherapy treatment, with only 59% of patients completing more than six cycles.

Despite our lower survival rates, yet those are still higher than the 15% 5-year relapse free survival reported for a group of young patients (≤14 years) with the diagnosis of Ewing's sarcoma in other anatomic locations treated in our institute between 1980 and 1993 (personal communication). Similarly, the IESS reported a higher median survival for head and neck location in comparison to Ewing's sarcoma in other anatomic sites.[1]

The only prognostic factor significantly affecting both DFS and OS in this study was the response to chemotherapy. This finding is in agreement with the recent articles that emphasize the importance of the initial response to chemotherapy treatment as the only significant independent predictor of survival for Ewing's sarcoma patients.[14] It should be noted,

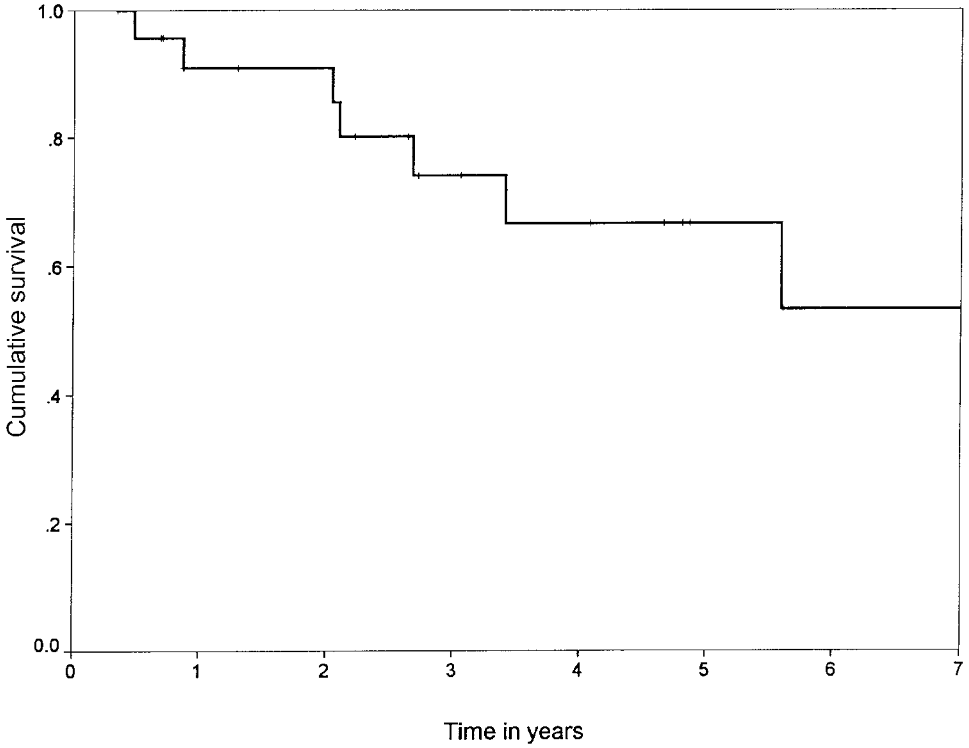

**Fig. 1.** *(a) Overall survival for the whole group.*

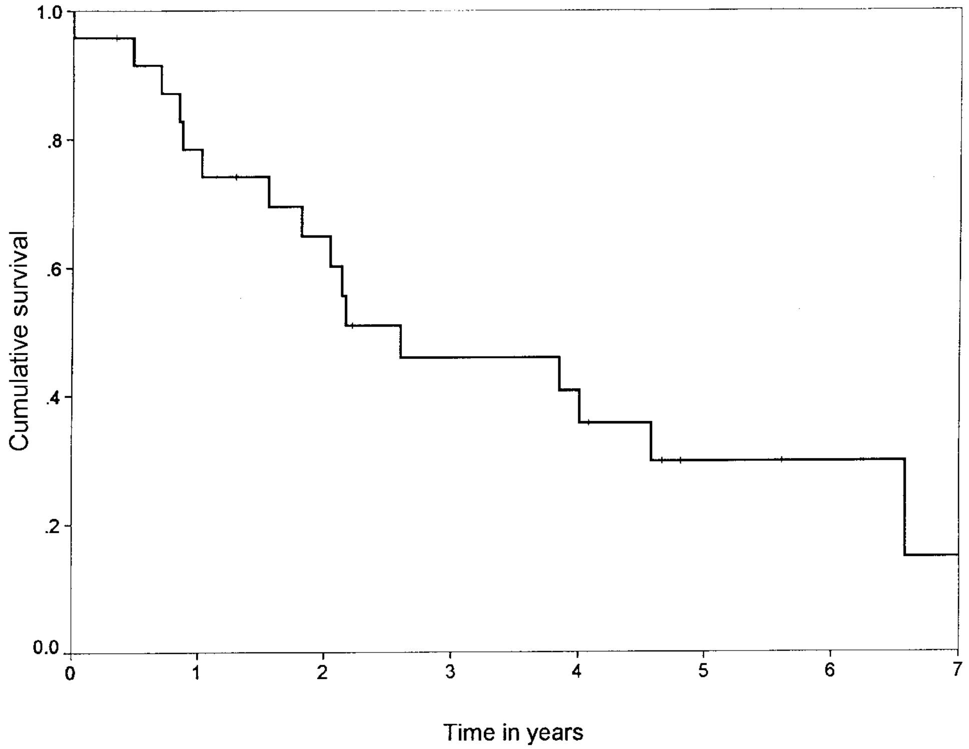

**Fig. 1.** *(b) Disease-free survival for the whole group.*

however, that the power of subanalysis in this study was low due to the small number of patients.

In the early phase of the present study, patients were treated with the usual VAC combination regimens, however, starting in 1995, with the introduction of more aggressive chemotherapy, we are now using VACA alternating with I/E (ifosfamide, etoposide) for a total of 52 weeks. This new alternating chemotherapy combination regimen has already yielded a statistically significant improvement in DFS when compared to the standard VACA alone in a phase III randomized study.[15] In conclusion, Ewing's sarcoma of the head and neck region carries a slightly better prognosis than that in other sites. Multimodality therapy consisting of an initial biopsy, aggressive combination chemotherapy and localized radiotherapy is the usual treatment of choice and could result in long-term disease-free survival. Radical surgery may be performed for local control in certain anatomic locations (e.g. mandibular and skull lesions), however, this needs to be followed by an adequate reconstructive surgery. The response to chemotherapy treatment remains one of the most important prognostic factors affecting the DFS as well as the OS of patients with Ewing's sarcoma of the head and neck region.

### References

1 Seigal GP, Oliver WR, Reinus WR, *et al.* Primary Ewing's sarcoma involving the bones of the head and neck, *Cancer* 1987; 60:2829–40.

2 Watanabe H, Tsubokawa T, Katayama Y, Koyama S, Nakamura S. Primary Ewing's sarcoma of the temporal bone. *Surg Neurol* 1992; 37:54–8.

3 Alvarez A, Schut L, Bruce D. Localized primary intracranial Ewing's sarcoma of the orbital roof. Case report. *J Neurosurg* 1979; 50:811–3.

4 O'Connell JE, Calder C, Raafat F, Proops D. Ewing's sarcoma of the retropharynx. *The Journal of Laryngology and Otology* 1994; 108:363–6.

5 Hadfield MG, Luo VY, Williams RL, Ward JD, Russo CP. Ewing's sarcoma of the skull in an infant. *Pediatr Neurosurg* 1996; 25:100–4.

6 Falk S, Alpert M. Five year survival of patients with Ewing's sarcoma. *Surg Gynecol Obst* 1967; 124:319–24.

7 Dahlin DC, Coventry MB, Scanlon PW. Ewing's sarcoma: a critical analysis of 165 cases. *J Bone Joint Surg* 1961; 43A:185–92.

8 Posnick JC, Louie G, Zuker R, Weitzman S. Ewing's sarcoma: primary involvement of the zygoma undergoing resection and immediate reconstruction. *Plast Reconstr Surg* 1992; 89:956–61.

9 Fiorillo A, Tranfa F, Canale G, *et al.* Primary Ewing's sarcoma of the maxilla, a rare and curable localization: report of two new cases, successfully treated by radiotherapy and systemic chemotherapy. *Cancer Letters* 1996; 103:177–82.

10 Zenke K, Hatakeyama T, Hashimoto H, Sakaki S, Manabe K. Primary Ewing's sarcoma of the occiptal bone—Case report. *Neurol Med Chir* 1994; 34:246–50.

11 Mishra HB, Haran RP, Joseph T, Chandi SM. Primary Ewing's sarcoma of the skull. A report of two cases. *Br J Neurosurg* 1993; 7:683–6.

12 Razek A, Perez CA, Tefft M, *et al.* Intergroup Ewing's Sarcoma Study. Local control related to radiation dose, volume, and site of primary lesion in Ewing's sarcoma. *Cancer* 1980; 46:516–21.

13 Burnet NG, Bliss JM, Harmer CL. The impact of radiotherapy dose on local control of Ewing's sarcoma of bone. *Sarcoma* 1997; 1:31–8.

14 Delepine HC, Alkallaf S, Brun B, Markowska B, Desbois JC. Is age a prognostic factor in localised Ewing's sarcoma treated by multidrug regimens and systematic surgery? *Proceedings of the American Society of Clinical Oncology (ASCO), 33rd Annual Meeting*, abstract # 1890, p. 525a, 1997.

15 Grier HE, Krailo M, *et al.* Improved outcome in non-metastatic Ewing's sarcoma (EWS) and PNET of bone with the addition of ifosfamide and etoposide to vincristine, adriamycin, cyclophosphamide and actinomycin: A Children's Cancer Group (CCG) and Pediatric Oncology Group (POG) Report. *Proc ASCO* 1994; 13:421.

## References

[PDF_00375] Alvarez-Berdecia A., Schut L., Bruce D. A. (1979). Localized primary intracranial Ewing's sarcoma of the orbital roof. Case report.. J Neurosurg.

[PDF_00408] Burnet N. G., Bliss J. M., Harmer C. L. (1997). The Impact of Radiotherapy Dose on Local Control of Ewing's Sarcoma of Bone.. Sarcoma.

[PDF_00386] DAHLIN D. C., COVENTRY M. B., SCANLON P. W. (1961). Ewing's sarcoma. A critical analysis of 165 cases.. J Bone Joint Surg Am.

[PDF_00384] Falk S., Alpert M. (1967). Five-year survival of patients with Ewing's sarcoma.. Surg Gynecol Obstet.

[PDF_00393] Fiorillo A., Tranfa F., Canale G., Fariello I., D'Amore R., De Chiara C., Vassallo P., Muto P., De Rosa G., Bonavolonta G. (1996). Primary Ewing's sarcoma of the maxilla, a rare and curable localization: report of two new cases, successfully treated by radiotherapy and systemic chemotherapy.. Cancer Lett.

[PDF_00381] Hadfield M. G., Luo V. Y., Williams R. L., Ward J. D., Russo C. P. (1996). Ewing's sarcoma of the skull in an infant. A case report and review.. Pediatr Neurosurg.

[PDF_00401] Mishra H. B., Haran R. P., Joseph T., Chandi S. M. (1993). Primary Ewing's sarcoma of the skull. A report of two cases.. Br J Neurosurg.

[PDF_00378] O'Connell J. E., Calder C., Raafat F., Proops D. (1994). Ewing's sarcoma of the retropharynx.. J Laryngol Otol.

[PDF_00389] Posnick J. C., Louie G., Zuker R., Weitzman S. (1992). Ewing's sarcoma: primary involvement of the zygoma undergoing resection and immediate reconstruction.. Plast Reconstr Surg.

[PDF_00404] Razek A., Perez C. A., Tefft M., Nesbit M., Vietti T., Burgert E. O., Kissane J., Pritchard D. J., Gehan E. A. (1980). Intergroup Ewing's Sarcoma Study: local control related to radiation dose, volume, and site of primary lesion in Ewing's sarcoma.. Cancer.

[PDF_00369] Siegal G. P., Oliver W. R., Reinus W. R., Gilula L. A., Foulkes M. A., Kissane J. M., Askin F. B. (1987). Primary Ewing's sarcoma involving the bones of the head and neck.. Cancer.

[PDF_00372] Watanabe H., Tsubokawa T., Katayama Y., Koyama S., Nakamura S. (1992). Primary Ewing's sarcoma of the temporal bone.. Surg Neurol.

[PDF_00398] Zenke K., Hatakeyama T., Hashimoto H., Sakaki S., Manabe K. (1994). Primary Ewing's sarcoma of the occipital bone--case report.. Neurol Med Chir (Tokyo).

